# Renal lymphangiectasia: know it in order to diagnose it

**DOI:** 10.1590/0100-3984.2015.0025

**Published:** 2016

**Authors:** Andréa Farias de Melo Leite, Bruna Venturieri, Rosana Gonçalves de Araújo, Eduardo Just Costa e Silva, Jorge Elias Junior

**Affiliations:** 1 Instituto de Medicina Integral Professor Fernando Figueira de Pernambuco (IMIP), Recife, PE, Brazil.; 2 Faculdade de Medicina de Ribeirão Preto da Universidade de São Paulo (FMRP-USP), Ribeirão Preto, SP, Brazil.

**Figure 1:**
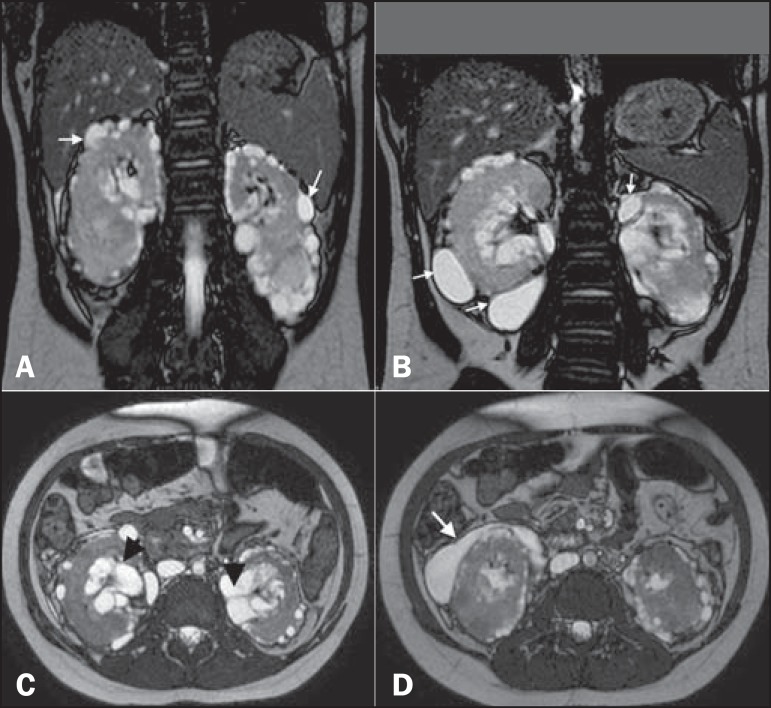
**A:** Coronal T2-weighted MRI sequence showing a loss of corticomedullary differentiation in both kidneys and multiple cystic lesions, with thin walls, located in the cortex (arrows). **B:** Cystic formations in the subcapsular cortex (arrows). **C:** Axial T2-weighted MRI sequence showing cysts located in the renal sinuses (arrowheads) and perinephric spaces, simulating pelvic dilatation. **D:** The same images simulating cystic collections in the subcapsular cortex (arrow).

Dear Editor,

Here, we report the case of a 9-year-old girl with hyperparathyroidism. Ultrasound showed renal cysts and increased echogenicity of the parenchyma in both kidneys. The diagnostic hypothesis was hyperparathyroidism secondary to chronic/polycystic kidney disease. The patient presented with gradually worsening kidney function and hypertension, and new imaging scans were requested. The ultrasound showed anechoic, multiloculated images in the pyelocaliceal region of both kidneys, and perirenal, subcapsular cysts. A computed tomography (CT) scan was acquired, although no contrast agent was used, which precluded an accurate characterization. Nevertheless, the CT scan revealed changes similar to those observed on ultrasound. We also performed magnetic resonance imaging (MRI), which showed pyelocaliceal, perirenal cysts, with altered intensity of the signal of the renal parenchyma and loss of corticomedullary differentiation (Figure 1A), confirming, in conjunction with the clinical and biochemical data, the diagnosis of renal lymphangiectasia (RL).

RL is a rare benign disease that occurs because of miscommunication between the renal lymphatic drainage system and the retroperitoneal lymphatic system^(1)^. As a result, there is accumulation of lymph in the renal lymph ducts, making them ectatic and forming simple or multiloculated, typically asymmetric and bilateral, collections in the pyelocaliceal, perinephric, or parenchymal regions, although, in some cases, only a part of one kidney is affected (Figure 1-B,C). There is no predilection for a given gender or age group. As of 2005, only 40 cases had been described^(1,2)^. In most cases, RL is an incidental finding, with or without signs and symptoms of pain, increased abdominal volume, hematuria, ascites, edema of the lower limbs, hypertension, erythrocytosis with renal vein thrombosis, and, rarely, chyluria^(3)^. Such manifestations can be explained by the distention of the renal fascia and compression of the renal parenchyma by cysts, fistulization to the pelvic cavity, and changes in the renin-angiotensin system^(2-4)^. In rare cases, chronic kidney disease has been reported^(5)^. To our knowledge, there have been no specific reports of clinical evolution to hyperparathyroidism, although a relationship with chronic kidney disease can be assumed. A CT scan can reveal expansive perirenal formations with fluid attenuation, bounded by the renal fascia, that conform to (and do not invade) the adjacent structures. Those formations can compress the kidney cortex, expand the sinus and distort the calyceal system. In some cases, small, predominantly peripheral, hypodense collections can be seen, with attenuation values of 0-15 HU^(3)^. There may be thickening of the renal fasciae and retroperitoneal collections crossing the midline at the level of the renal hilum. After the administration of iodine contrast, there is no enhancement of the collections or of the walls of the cystic formations^(6)^. In MRI, the cysts exhibit a low signal on T1-weighted sequences-although the signal strength can be increased if there is bleeding^(6)^-and a more intense signal on T2-weighted sequences, without enhancement. In addition, RL can be diagnosed on MRI scans by identifying perirenal lymphatic collections with inversion of the corticomedullary signal intensity^(1,4)^, as depicted in Figure 1-B,C,D. To suggest a diagnosis of RL, as well as to devise a treatment strategy and to prevent complications, it is essential to understand the radiological aspects of the disease and to differentiate it from other conditions that mimic cystic kidney disease. Although the combination of RL and renal failure is rare, knowledge of that association is also important to prevent comorbid conditions that can evolve with this complication, such as obesity and high blood pressure.
